# Multi-Currency Integrated Serial Number Recognition Model of Images Acquired by Banknote Counters

**DOI:** 10.3390/s22228612

**Published:** 2022-11-08

**Authors:** Woohyuk Jang, Chaewon Lee, Dae Sik Jeong, Kunyoung Lee, Eui Chul Lee

**Affiliations:** 1 Department of Computer Science, Graduate School, Sangmyung University, Hongjimun 2-gil 20, Jongno-gu, Seoul 03016, Republic of Korea; 2 Department of AI & Informatics, Graduate School, Sangmyung University, Hongjimun 2-gil 20, Jongno-gu, Seoul 03016, Republic of Korea; 3 Division of Software Convergence, Sangmyung University, Hongjimun 2-gil 20, Jongno-gu, Seoul 03016, Republic of Korea; 4 Department of Human-Centered Artificial Intelligence, Sangmyung University, Hongjimun 2-gil 20, Jongno-gu, Seoul 03016, Republic of Korea

**Keywords:** deep learning, image processing, image recognition, optical character recognition, pattern recognition

## Abstract

The objective of this study was to establish an automated system for the recognition of banknote serial numbers by developing a deep learning (DL)-based optical character recognition framework. An integrated serial number recognition model for the banknotes of four countries (South Korea (KRW), the United States (USD), India (INR), and Japan (JPY)) was developed. One-channel image data obtained from banknote counters were used in this study. The dataset used for the multi-currency integrated serial number recognition contains about 150,000 images. The class imbalance problem and model accuracy were improved through data augmentation based on geometric transforms that consider the range of errors that occur when a bill is inserted into the counter. In addition, by fine-tuning the recognition network, it was confirmed that the performance was improved when the serial numbers of the banknotes of four countries were recognized instead of the serial number of a banknote from each country from a single-currency dataset, and the generalization performance was improved by training the model to recognize the diverse serial numbers of multiple currencies. Therefore, the proposed method shows that real-time processing of less than 30 ms per image and character recognition with 99.99% accuracy are possible, even though there is a tradeoff between inference speed and serial number recognition accuracy when data augmentation based on the characteristics of banknote counters and a 1-stage object detector for banknote serial number recognition is used.

## 1. Introduction

**Figure 1:**
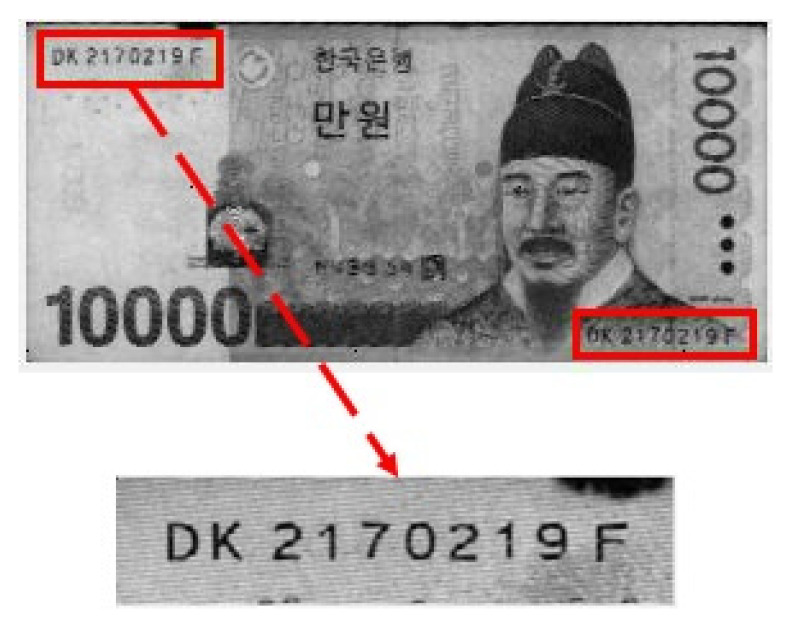
Example of a banknote serial number (KRW 10,000 bill).

Although electronic payment methods are increasing and the use of banknotes is on the decline worldwide, banknotes still play a significant role and are a major means of transactions and a secure store of wealth [1]. Although counting banknotes by hand is a common practice, to facilitate fast and accurate large-scale banknote transactions, the use of automated machines has become essential. These machines include banknote counters [2], coin counting machines [3], and automatic vending machines [4], in which money is inserted to purchase goods, as well as automated teller machines for deposits and withdrawals of banknotes. In the banking sector, devices such as banknote classifier machines, which are capable of completing large-scale transactions faster and more accurately than bank employees, have become essential for most banking transactions. Such devices perform complex functions such as banknote recognition, counterfeit status detection, and large-scale batch processing to satisfy the diverse requirements of banknote transactions. The major areas of banknote image analysis [5] include counterfeit banknote detection, serial number recognition, fitness classification, and banknote recognition. A banknote serial number, as shown in Figure 1, is engraved on each banknote in the process of production and is a unique alphanumerical identifier typically consisting of 9 to 11 digits [6]. When the unique serial number of a banknote is recognized and recorded, the source and circulation route of the banknote can be traced. This method is effective for the detection of counterfeit banknotes.

**Figure 2:**
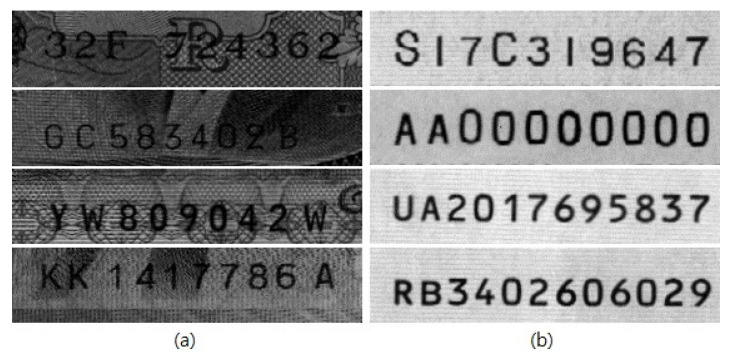
Examples of the backgrounds of serial number regions: (**a**) serial numbers with complex background patterns; (**b**) serial numbers with clean background patterns.

Banknote serial number recognition methods are based on optical character recognition (OCR) algorithms, which have been developed and applied in other areas, such as license plate recognition [7] and street number recognition based on Street View images [8]. In contrast to other fields adopting OCR, banknote serial number recognition requires a higher standard of accuracy because even small errors can result in large financial losses. Additionally, real-time detection is important for banknote service terminals, and the recognition process must satisfy transaction time requirements [9]. In general, the serial number recognition process for banknotes is divided into two steps. In the first step (preprocessing), the serial number region is extracted as the region of interest (ROI) containing the serial number from the banknote image captured by contact image sensors (CISs). In the extracted serial number region, segmentation is performed for each alphanumeric character. The segmentation process involves binarization and horizontal–vertical projection. In the second step (the character recognition process), feature extraction and classification are performed. In the feature extraction stage, the unique features of each alphanumeric character are extracted. These features include handcrafted features such as Gabor features and histogram of oriented gradients (HOG) features. In the classification part, each alphanumeric character is classified using the extracted features. The classifiers that can be used in this process include k-nearest neighbors, neural networks, and support vector machines (SVMs). Among them, SVMs are commonly used because they are less susceptible to overfitting and easier to use than neural networks. They also obtain a good real-time recognition rate for embedded systems installed in terminals. These algorithms can be utilized to obtain results with high accuracy for serial number recognition. In the ideal situation of banknotes with clean background patterns in the serial number region (Figure 2b), the recognition rate is excellent, but in practice, many banknotes do not have clean background patterns, as shown in Figure 2a. Banknotes with complex background patterns mainly include those with no clear boundary or outline (hindering character recognition), those with a small difference in pixel intensity between the background and characters, and those with similar styles for the background and serial number. Such notes cause difficulties in feature extraction. Additionally, the recognition accuracy is reduced by dirt, creases, and damage to the serial number region. However, with the development of artificial intelligence techniques, new methods have been introduced, and a deep learning (DL)-based approach has been developed to address the limitations of the existing algorithms and improve performance. In this study, the serial number region images of banknotes in multiple currencies (KRW, USD, INR, and JPY) are used as inputs for a convolutional neural network (CNN) to create an object detection model that localizes and classifies serial numbers for recognition. We use this approach to propose a multi-currency integrated serial number recognition model that is based on a DL 1-stage detector structure and is robust against various fonts and types of background noise. The contribution points of our proposed method are as follows.

1. The proposed method is a 1-stage method that simultaneously performs character region detection and classification instead of only character recognition.
2. The proposed method achieves state-of-the-art performance on both detection and recognition tasks because the 1-stage object detector is optimized for serial number recognition and the data are augmented according to the characteristics of banknote counters.
3. It achieves the highest serial number recognition performance compared with previous methods despite the increase in classes and the additional detection task.
4. The recognition model trained using a multi-currency dataset performed better than the recognition model trained on only single-currency datasets, indicating that the multi-currency dataset improved the generalization performance of the recognition model.

The remainder of this paper is organized as follows. Section 2 presents a literature review of serial number recognition. Section 3 describes the data collection and preprocessing steps and presents the proposed model. Section 4 presents the results obtained using the integrated serial number recognition model. In Section 5, the paper is summarized, and the conclusions are drawn.

## 2. Related Work

### 2.1. Handcrafted Feature Extraction Approach

Statistical recognition methods perform recognition using the feature vectors of each serial number character. Feature vectors are extracted from input images, the model is trained, and the probability distributions of the extracted feature vectors are obtained to separate the feature vector space. In these methods, the recognition results are affected by the definitions of the features of each serial number and the criteria for extracting feature vectors; thus, accurate definitions are crucial.

Zhao et al. [10] employed gray-level transformations from RGB images obtained using a charge-coupled device camera, binarization, and slope correction for preprocessing. Next, serial numbers were segmented using a vertical projection segmentation method. For the classifier, a genetic algorithm–backpropagation artificial neural network (GA-BP ANN) was used, and bilinear interpolation was used to address the problem of slow or no network convergence when samples of different sizes were used. The GA was used to set the node weights of the ANN. The accuracy of the results obtained using the GA-BP ANN was 95%, whereas that for a BP ANN was 82%. Ebrahimzadeh et al. [11] segmented the digits for recognition and extracted HOG features in the preprocessing step. Then, an SVM was employed using four different kernels—linear, polynomial, radial basis function (RBF), and sigmoid. Among these, the linear kernel exhibited the best performance, with an accuracy of 97.25%. This structure is efficient and useful for real-time application. Feng et al. [12] focused on RenMinBi (RMB) serial numbers. A variety of feature extraction methods use gradient direction features, Gabor features, and LeNet5 network-based features. For classifiers, an SVM with a linear discriminant function, quadratic discriminant function, and modified quadratic discriminant function was used. A multivariate normal distribution was assumed, and classification was performed using Bayes’ theorem. Using these methods, serial number recognition was performed. In another study by Feng et al. [13], points of interest were extracted using the difference of Gaussians to segment the serial number region. Then, the k-means clustering algorithm was used to remove redundant points of interest, and the key points were clustered to generate a set of local image parts for the serial number. A classification accuracy of 99.33% was achieved using SVM models with linear and RBF kernels. 

In addition, other OCR algorithms, such as machine learning algorithms for scene text recognition and other methods, can be used to recognize banknote serial numbers. To recognize scene text in different languages, Tian et al. [14] extended the conventional HOG and proposed two new feature descriptors—the co-occurrence HOG (Co-HOG), which extracts spatial information by calculating the co-occurrence frequency of gradients for neighboring pixels, and the convolutional Co-HOG (ConvCo-HOG), which uses a convolutional strategy to extract Co-HOG features for all possible distributions of the co-occurrence structure. In addition, the dimensionality was reduced using principal component analysis, and scene text characters were recognized using a linear SVM. Zhou et al. proposed a method [15] for extracting characters from a serial number and removing background and noise using a hybrid binarization (HybridB) algorithm. This algorithm combines the binary algorithm based on the cumulative statistics of gray histograms, which performs projection on the horizontal and vertical character region of the serial number, and the binary algorithm based on neighborhood second expansion of gray ratios, which utilizes the four-way neighborhood information of each pixel. In addition, an adaptive character extraction algorithm was proposed to extract characters.

### 2.2. Deep Learning-Based Approach

DL methods employing CNNs have achieved good performance in various vision-based applications, and these methods exhibit considerable potential for serial number recognition. In DL, training is performed automatically using input data obtained using a learned feature extraction method rather than a handcrafted feature extraction method. Alwzwazy et al. [16] performed DL-based character recognition for Arabic digits, which is more difficult than the recognition of general alphanumeric patterns. The input images were the segmented Arabic digits, and because these were passed to the designed architecture, the images were preprocessed to make them 64 × 64 pixels in size. The DL architecture consisted of a convolutional layer, a max-pooling layer, and a fully connected layer. Classification was performed using the softmax activation function. This model achieved an accuracy of 95.7%. Boufenar et al. [17] performed DL-based character recognition with OAHCDB-40 [18] and AHCD, which are two datasets of segmented Arabic characters. The image size was 227 × 227 pixels, and the AlexNet architecture was used for model training. Three different training methods were applied—training from scratch, CNN fine-tuning, and the use of the CNN as a fixed feature extractor. Training from scratch achieved an accuracy of 100%. Zhao et al. [19] employed horizontal and vertical projections to determine the left- and right-side positions of each character as well as the window size of the character. Characters were extracted by a sliding window method and, using a simple CNN structure, a recognition accuracy of 99.99% and a recognition time of 5 ms were achieved using a DM642 chip. Wang et al. [20] performed preprocessing involving skew correction and the segmentation of each character on grayscale images of banknotes scanned using CISs. Image data with each extracted character were used, and the convolutional and pooling layers were replaced with dilated convolution in the CNN, which reduced its computational cost. In addition, the quantitative neural network-based method quantizes the weight parameters to an integer power of two, which can significantly reduce the learning time. In an experiment, the accuracy was increased to 99.89%, and a recognition time of less than 0.1 ms was achieved. Jang et al. [21] proposed a serial number recognition method for Indian banknotes, which have a unique font and complex background. A de-skewing process was performed on the images obtained for serial number recognition, the serial number region was cropped to extract each character, and a data shift was applied as data augmentation to prevent overfitting. The DL structure was composed of deep/shallow layers and heavy/light kernels; thus, four different models were designed: (1) deep/heavy, (2) deep/light, (3) shallow/heavy, and (4) shallow/light. With the shallow/light structure, an accuracy of 99.92% was obtained, and it was demonstrated that the DL character recognition method could recognize serial numbers in banknotes with complex backgrounds and characters with unusual fonts. 

In addition, other OCR algorithms and related methods can be applied to banknote serial number recognition. Namysl et al. [22] introduced a lexicon-free OCR system. To recognize complex scene text, synthetic data were generated using a corpora and 2000 fonts. The DL model used the convolutional recurrent neural network structure, which is a hybrid method combining a CNN and long short-term memory (LSTM) and offers excellent feature extraction performance. The proposed OCR system achieved a higher accuracy than an open-source engine; it represents an application that combines a CNN and LSTM. Caldeira et al. [23] proposed an OCR system to extract the printed identification numbers of steel coils. The ROI of the coil identification number was extracted from an image obtained using a fixed camera. Then, to extract each character, the background was separated using four filters—the top hat, homomorphic, LogAbout, and Otsu filters. Each character was segmented by extracting the connected components from the background-separated binary images, and AlexNet, LeNet-5, and CIFAR models were used. Among these models, the LeNet-5 model yielded the best results, with an accuracy of 99.68%. Gang et al. [24] proposed a model for recognizing characters printed on components mounted on printed circuit boards. Data were collected automatically from inspection machines. Using the software used in the inspection process, characters were separated and labeled from the printed strings. In addition, data augmentation techniques, illumination, rotation, size, and noise were varied, and for the DL model, ResNet [25] and EfficientNet [26] were used to achieve a top-5 accuracy of 99.965%.

## 3. Materials and Methods

**Figure 3:**
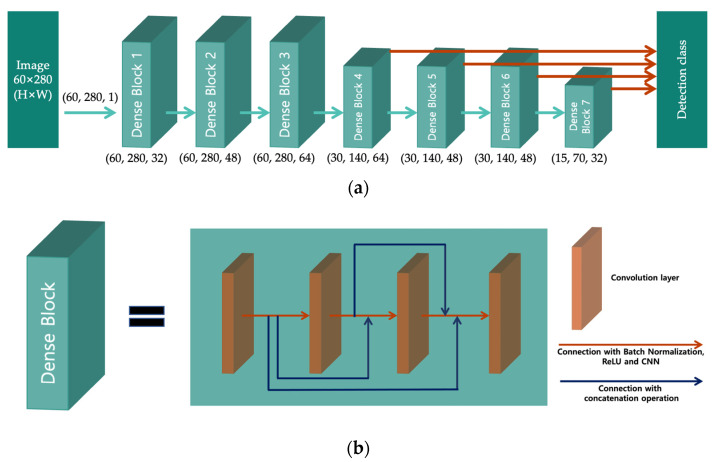
Single shot multi-box detector (SSD) based multi-currency integrated serial number recognition model: (**a**) proposed model; (**b**) dense block comprising the backbone of the proposed model.

This section presents a detailed description of the development of the proposed model. Our method consists of the following four steps: data acquisition, preprocessing, DL model training, and multi-currency banknote serial number recognition. The proposed method is based on a single-shot multi-box detector (SSD), which is a 1-stage object detector [27]. The proposed method simultaneously performs detection and classification tasks, unlike existing methods that perform only character classification and do not detect character regions. The backbone of the proposed method is a CNN, which is a dense block-based network proposed by Huang et al. [28]. The proposed method is an SSD model in which the input size and backbone network are set according to the serial number recognition task, as shown in Figure 3. Experiments were conducted to determine the optimal SSD structure and hyperparameter settings for serial number recognition, and the process is described in Section 3.1 and Section 3.2.

### 3.1. Data Acquisition and Preprocessing

**Figure 4:**
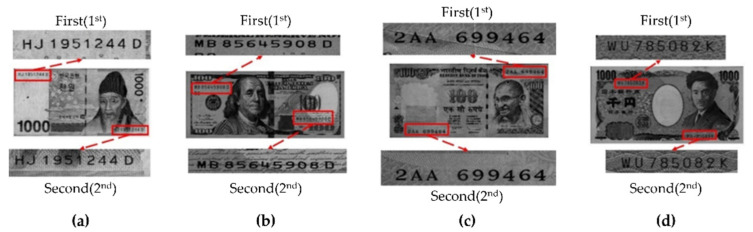
Example of ROI selections for different positions of the banknote serial number: (**a**) KRW; (**b**) USD; (**c**) INR; (**d**) JPY.

The data used in this study were acquired using a banknote counter [29] with a CIS (Canon Inc., Otaku, Japan). The CIS sensor-based banknote counter acquires approximately 16 frames per second, and the image resolution is 200 DPI. The acquired data are images of various types of banknotes from four countries (South Korea, the United States, India, and Japan). For Korean currency (KRW), denominations of 1000, 5000, 10,000, and 50,000 won were used. For US currency (USD), denominations of 1, 2, 5, 10, 20, 50, and 100 US dollars were used. For Indian currency (INR), denominations of 10, 20, 50, 100, 200, 500, and 2000 rupees were used. For Japanese currency (JPY), denominations of 1000, 2000, 5000, and 10,000 yen were used. The data acquired using the banknote counter were in a file in bag-of-features format with ROI information for serial number region. Using this file, the serial number regions were cropped and stored as 1-channel bitmap images (280 × 60). Because the same serial number can be found in two positions on each bill, the images were saved by assigning a separate ROI for each case. Although there are differences among the banknotes of different countries, the serial numbers are mainly located in the top left and bottom right corners. The serial number in the top left was marked as “first”, and the serial number in the bottom right was marked as “second”. Figure 4 shows an example of ROI selection.

**Figure 5:**
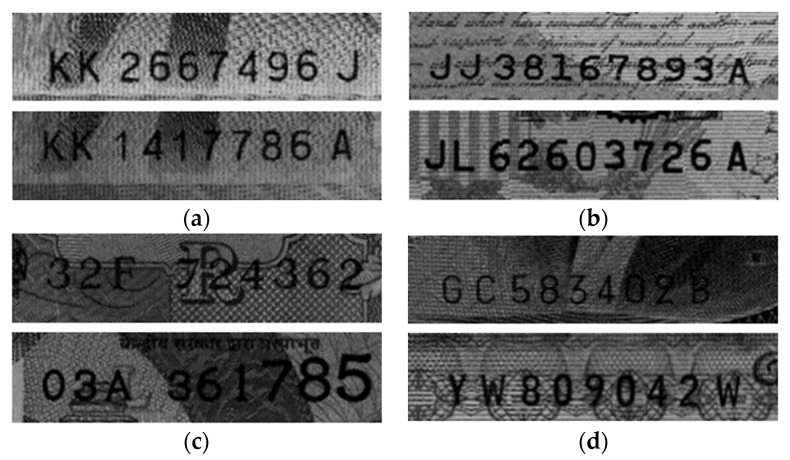
Examples of cropped ROI images for each currency: (**a**) KRW; (**b**) USD; (**c**) INR; (**d**) JPY.

An examination of the cropped images of the serial number regions of the banknotes revealed that, in general, the national currencies have the following characteristics. Korean and US banknotes have serial numbers with similar sizes. However, in Indian currency, different types of banknotes have serial numbers of different sizes. In addition, the serial numbers of US banknotes uniquely contain the character “☆”. In Japanese banknotes, the serial numbers are unclear because the pixel intensities of the background and serial numbers are similar. The images were manually labeled according to the bounding box for each character and the predefined class ID. Figure 5 shows an example of cropped images for each national currency. 

**Figure 6:**
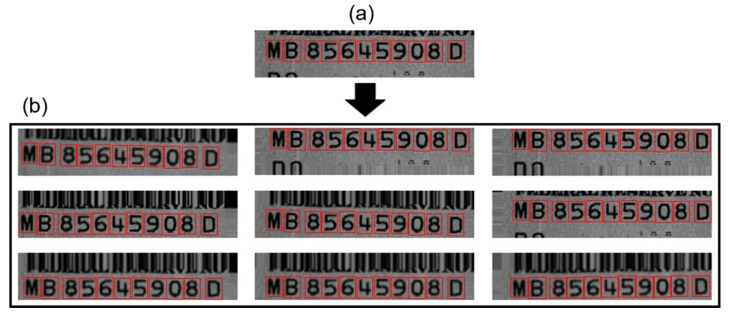
Example of image data augmentation for banknote image data. Red boxes indicate the ground truth. (**a**) Original image; (**b**) images obtained from data augmentation.

When a note is inserted into the banknote counter, slight rotation and translation occur. Additionally, banknotes with frequent circulation are subjected to soiling and creases. To represent similar situations when performing data augmentation, the data were augmented 10-fold by applying mixed techniques of rotation, translation, and blur. The rotation is defined by Equation (1), and a range of –1° to +1° was applied with reference to the image center, considering that the CIS sensor acquires images aligned with the sensor’s image plane. Translation was specified in eight directions—up, down, left, right, and four diagonal directions—using Equation (2). For the padding, the pixel values at the edge were used for filling. Finally, Gaussian blur, expressed in Equation (3), was used [30]. Figure 6 shows an example of the augmentation applied to the banknote image data.

(1) $$\left[\begin{array}{c} x^{\prime }\\ y^{\prime }\\ 1 \end{array}\right]=\left[\begin{array}{c} \cos\theta \;\sin\theta \;0\\ -\sin\theta \;\cos\theta \;0\\ \;\;\;0\;\;\;\;\;\;\;\;\;0\;\;\;\;\;\;\;1 \end{array}\right]\left[\begin{array}{c} x\\ y\\ 1 \end{array}\right]$$

(2) $$\left[\begin{array}{c} x^{\prime }\\ y^{\prime }\\ 1 \end{array}\right]=\left[\begin{array}{c} 1\;0\;\Delta x\\ 0\;1\;\Delta y\\ 0\;0\;\;\;1 \end{array}\right]\left[\begin{array}{c} x\\ y\\ 1 \end{array}\right]$$

(3) $$I\left(x,y;\sigma \right)=\frac{1}{2\pi \sigma^{2}}exp^{-\left(x^{2}+y^{2}\right)/2\sigma^{2}}$$

The composition of the datasets was as follows. The first and second ROI images and labels were divided into training, validation, and test sets and then pooled to form a multi-currency dataset. For the KRW, USD, INR, and JPY banknotes, the dataset composition was as follows: 70% for the training set, 15% for the validation set, and 15% for the test set. Table 1 presents the number of banknote data for each currency.

### 3.2. Model Architecture

**Figure 7:**
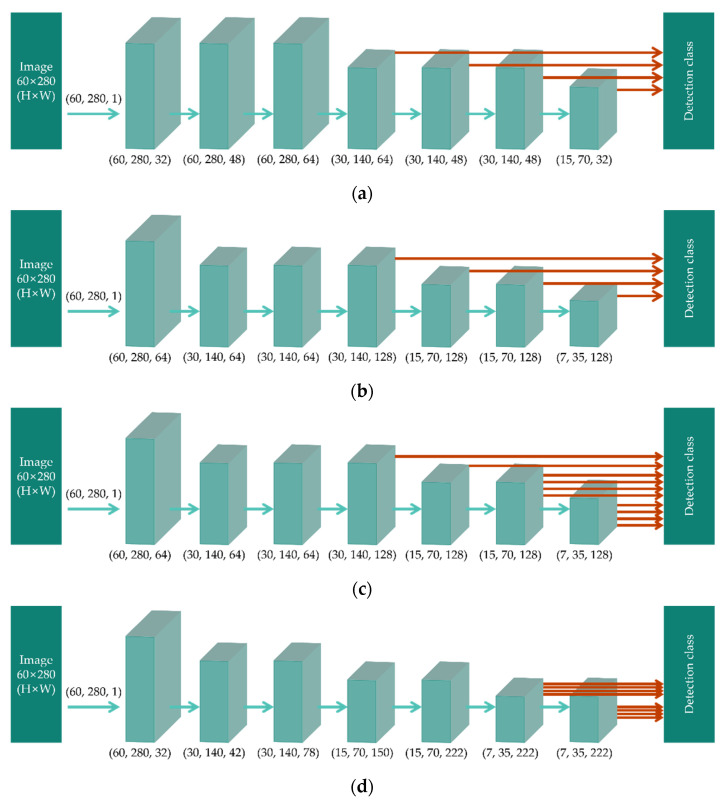
Modified architectures: (**a**) Version 1; (**b**) Version 2; (**c**) Version 3; (**d**) Version 4.

This section describes the model structure used in this study for multi-currency integrated serial number recognition and discusses the difference between the proposed and existing methods. In this study, four architectures were designed to identify the optimal architecture for multi-currency banknote serial number recognition. Figure 7 shows the structures of the four architectures. In the proposed model (Version 1), classification is performed using a high-level feature map with good feature extraction performance. In Version 2, pooling layers are added, which can reduce the training time; additionally, duplicate features are removed, and only important information is transferred. In Version 3, multiple classifier layers are added to allow the detection and recognition of small banknote serial numbers. Finally, in Version 4, DenseNet [29], which uses dense block and a transition structure, is employed. DenseNet has a structure that concatenates the feature maps of all layers. In this structure, features extracted from preceding layers can be continuously used, and the information flow is maximized. Additionally, to maximize feature extraction, the classifier layers use the extracted features from a high-level feature map.

## 4. Experimental Results

**Figure 8:**
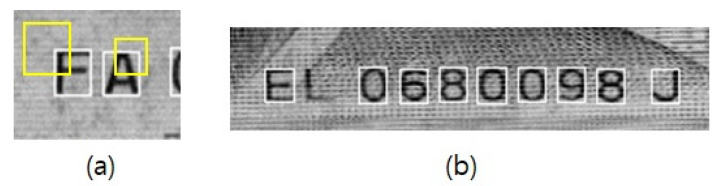
Examples of detection errors (yellow box: prediction; white box: ground truth): (**a**) first case; (**b**) second case.

In this section, the process of developing the proposed model for multi-currency banknote serial number recognition is described. First, to identify the optimal model structure for this task, all four model structures illustrated in Figure 7 were tested, and for each currency, four different DL models were created to evaluate the recognition accuracy. The model structure exhibiting the highest accuracy was selected to develop a model for integrated recognition of banknote serial numbers in multiple currencies. For an accurate and fair comparison of model performance, a pre-trained model was not used, and the convolutional layer hyperparameters were set using He initialization [31]. All experiments were implemented in Python and Keras and were performed on a PC equipped with an Intel^®^ Core (TM) i9-10900KF (3.70 GHz) CPU, an NVIDIA GeForce RTX2080 Ti GPU, and 64 GB of RAM. The output of the DL model includes the results of bounding-box detection and character classification. The errors were divided into detection errors and classification errors, and the sum of these errors was calculated to determine the total accuracy. A detection error refers to a failure in the detection of the bounding box. There are two cases of prediction failure. In the first case, the bounding box is detected, but the predicted location does not match the location of the ground truth but is elsewhere, leading to a small intersection over union [28]. In the second case, some parts of the serial number are not detected. Figure 8 illustrates examples of detection errors. The classification error refers to a failure in to predict the correct class. The accuracy values were calculated using Equations (4)–(6).

(4) $$\mathrm{detecton}\;\mathrm{accuracy}=\frac{\mathrm{number}\;\mathrm{of}\;\mathrm{correct}\;\mathrm{detections}}{\mathrm{total}\;\mathrm{number}\;\mathrm{of}\;\mathrm{detections}}$$

(5) $$\mathrm{recognition}\;\mathrm{accuracy}=\frac{\mathrm{number}\;\mathrm{of}\;\mathrm{correct}\;\mathrm{recognitions}}{\mathrm{total}\;\mathrm{number}\;\mathrm{of}\;\mathrm{recogntions}}$$

(6) $$\mathrm{total}\;\mathrm{accuracy}=\frac{\mathrm{number}\;\mathrm{of}\;\mathrm{correct}\;\mathrm{detections}\;\mathrm{and}\;\mathrm{recognitions}}{\mathrm{total}\;\mathrm{number}\;\mathrm{of}\;\mathrm{data}}\;$$

**Figure 9:**
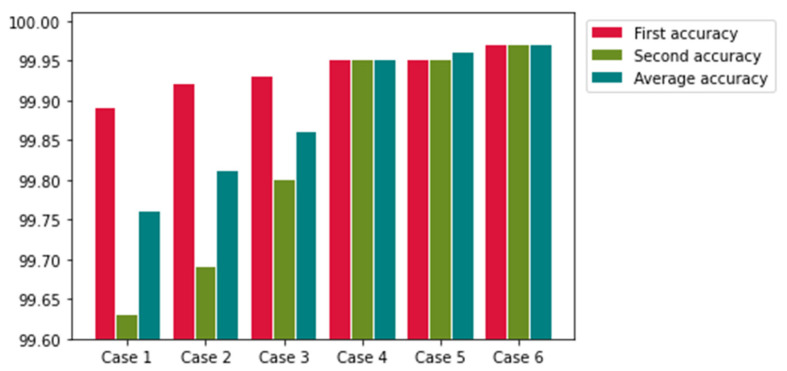
Graphical accuracy comparison for the result of Table 4.

In the SSD model, multiple confidence values and bounding boxes are created for an object in the training stage. Among the multiple bounding boxes, only the optimal bounding box of the object should be selected, and all the other boxes should be removed. This operation is performed by the non-maximum suppression algorithm [32]. Table 2 presents the recognition accuracy (detection and classification) and model processing time of the Version 1, Version 2, Version 3, and Version 4 models. Evidently, the Version 4 model yields the highest accuracy. Experiments were conducted to evaluate the performance improvements of the model obtained by scale and aspect ratio adjustments and annotation mistake modifications. Table 3 presents the different scales and aspect ratios of the anchor boxes applied to the banknotes of multiple currencies. Table 4 and Figure 9 present the accuracy of the recognition model when these values were applied. The aspect ratio and anchor box hyperparameters were defined based on the printed characteristics of the banknote serial numbers. Setting the hyperparameters based on these characteristics considerably reduced the detection and classification errors.

**Figure 10:**
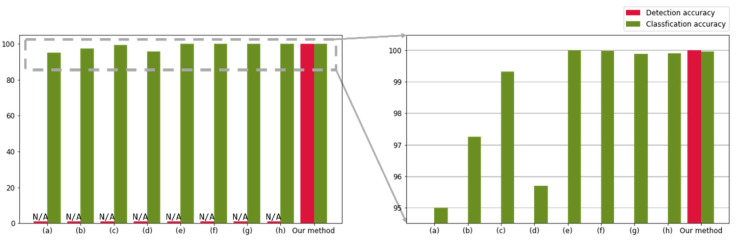
Graphical accuracy comparison for the result of Table 5 (Compared previous studies have marked “N/A” because only classification results exist without character region detection). (a) Zhao et al. [10]. (b) Ebrahimzadeh et al. [11]. (c) Feng et al. [13]. (d) Alwzwazy et al. [16]. (e) Boufenar et al. [17]. (f) Zhao et al. [19]. (g) Wang et al. [21]. (h) Jang et al. [22].

**Figure 11:**
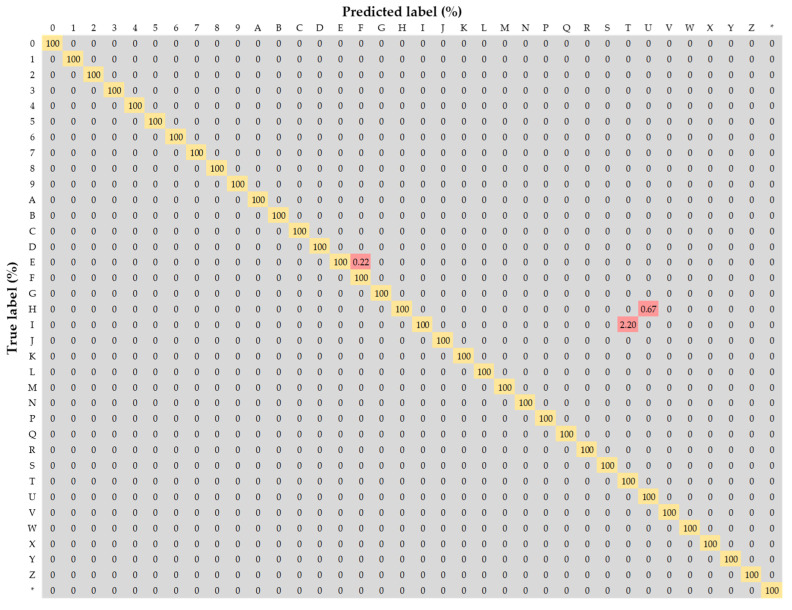
Multi-currency (KRW, USD, INR, JPY) confusion matrix for the test dataset.

Table 5 and Figure 10 compare the results of existing DL-based methods and the integrated recognition model for the banknotes of four currencies (KRW, USD, INR, and JPY). In previous studies, only classification was performed (not object detection) for the digits 0–9 and letters A–G. By contrast, in the proposed method, classification is performed over a total of 37 classes, including the digits 0–9, letters A–Z, and special character “☆” along with object detection. The comparison reveals that the proposed method is superior to the previously reported methods for banknote serial number recognition. Figure 11 presents a confusion matrix of the results of the multi-currency (KRW, USD, INR, and JPY) test set. The horizontal axis indicates the predicted label, and the vertical axis indicates the true label. Correct and incorrect predictions are shown in orange and red, respectively.

## 5. Discussion

**Figure 12:**
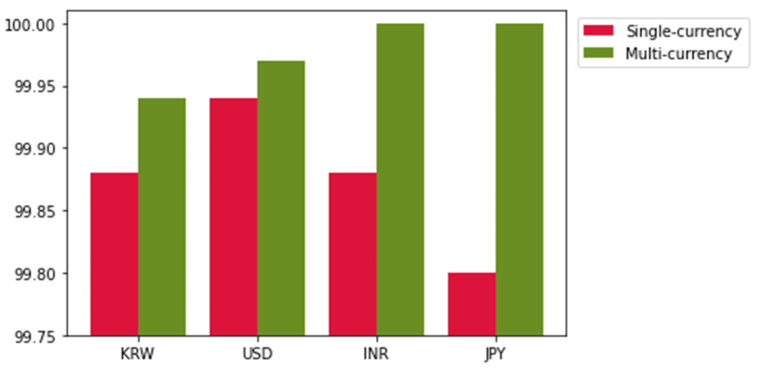
Graphical accuracy comparison for the result of Table 6.

In this study, an SSD-based multi-currency integrated serial number recognition model was developed as a 1-stage OCR model that considers both inference speed and OCR accuracy, unlike the 2-stage methods that detect character positions and classify characters. In contrast to these methods, the proposed method performs character detection and recognition for alphanumeric characters using an SSD-based 1-stage method. Despite the additional task of character recognition, the accuracy was confirmed to be state-of-the-art when compared with the accuracy of the existing methods. In addition, it is clear that the task assigned to the proposed method is more challenging than that of the existing methods—recognizing the banknotes of four countries using a single SSD model. When using our proposed multi-currency integrated serial number recognition model, a separate algorithm is not required to detect a character’s location, and the use of a large dataset for multiple countries improves the accuracy for the currency of a single country with an insufficient amount of data. Table 6 and Figure 12 compare the performance of models using only a single national currency dataset with the accuracy obtained when the model is trained on a large dataset containing four currencies. In the serial number recognition results for each national currency, it can be observed that using the data of multiple currencies is better than using only single-currency data. These results show that even if the font and size of the serial numbers differ among countries, the feature extraction is better when the model is trained with a large, integrated dataset. In addition, it was confirmed that the diversity and size of data were insufficient for training a model with high accuracy (approaching 100%) even when using a large dataset consisting of 40,000 or more samples for each country (see Table 1). In other words, the proposed method increased the diversity and number of data by integrating multiple currencies into one dataset, and a multi-currency integrated serial number recognition accuracy of 99.97% was obtained through model structure optimization and hyperparameters set according to the printing characteristics of the currencies. Finally, the proposed method is a serial number recognition model based on a fast SSD model with state-of-the-art character detection accuracy and can be applied to various tasks in the financial field, such as detecting counterfeit money with mismatched serial numbers or currency bill tracking. 

In the future, reducing the inference time to enable high-speed processing in embedded systems in banknote counters could be a major research topic. To this end, various DL techniques and frameworks could be utilized, such as knowledge distillation, lightweight CNNs, the Open Neural Network Exchange, and TensorFlow Lite. In the case of knowledge distillation, the teacher–student structure can drive learning, such that the student model achieves teacher-level accuracy, even though the student model has fewer parameters and is faster than the teacher model. In the case of lightweight CNNs, depth-wise separable convolution or grouped convolution can be used to improve the inference speed by reducing the number of floating point operations. Furthermore, by establishing a 1-stage object detector-based integrated banknote serial number recognition system for various currencies, such as Arabic and Russian Cyrillic banknotes, a state-of-the-art multi-currency integrated serial number recognition system could be developed, even if the serial number contains characters that are not from the Latin alphabet.

## 6. Conclusions

In this study, a state-of-the-art multi-currency integrated serial number recognition method was developed to obtain superior accuracy and inference speed. To identify the optimal model structure for banknote serial number recognition, CNN models with four different structures were designed, and a data augmentation technique based on the insertion of notes into the banknote counter was used. A performance comparison was performed using a dataset containing multiple currencies (KRW, USD, INR, and JPY) to determine the optimal 1-stage object detector-based architecture for multi-currency integrated serial number recognition. The results confirmed that the best representation was extracted from the layers of the last block. Therefore, the best architecture was designed using a loss function, and the model output was obtained from the last block’s feature map. Using the optimal model, an integrated serial number recognition model for the banknotes of the four countries was designed. To determine the optimal hyperparameter values, experiments were performed using different scales and aspect ratios. The proposed method, using hyperparameters optimized for the printing characteristics of serial numbers, achieved state-of-the-art performance, with 99.97% accuracy and real-time serial number detection (within 30 ms) for multi-currency serial number recognition. These results indicate the importance of adjusting the parameters for the creation of anchor boxes during the training stage. It was confirmed that the proposed CNN-based banknote serial number recognition method could achieve accurate banknote serial number recognition even in the presence of complex backgrounds.

## Figures and Tables

**Table 1:** Size of dataset for each currency.

Currency	Training	Validation	Test	Total
KRW	35,000	7500	7500	50,000
USD	37,724	8083	8094	53,901
INR	31,500	6750	6750	45,000
JPY	31,260	6695	6696	44,651
Total	97,080	26,624	21,832	145,536

**Table 2:** Model performance comparison of different architecture versions.

Version	1	2	3	4
Accuracy (%)	97.89	99.33	99.42	99.88
Number of detection errors	50	32	30	0
Number of classification errors	108	12	13	9
1st accuracy (%)	98.97	99.49	99.72	100
2nd accuracy (%)	96.81	99.17	99.12	99.75
Time (ms)	44	42	44	30

**Table 3:** Scale and aspect ratio of anchor boxes for use in the multi-currency (KRW, USD, INR, and JPY) recognition model.

Case	Scale	Aspect Ratio Set
1	[0.41, 0.45, 0.61, 0.65]	[0.81, 0.85, 0.88]
2	[0.30, 0.39, 0.47, 0.57]	[0.8, 0.9]
3	[0.33, 0.41, 0.55, 0.62]	[0.5, 0.65, 0.88, 1.0, 1.1, 1.25, 1.4]
4	[0.28, 0.36, 0.44, 0.52]	[0.5, 0.65, 0.88, 1.0, 1.1, 1.25, 1.4]
5	[0.28, 0.36, 0.44, 0.52]	[0.5, 0.65, 0.88, 1.0, 1.1, 1.25, 1.4]
6	[0.28, 0.36, 0.44, 0.52]	[0.5, 0.65, 0.88, 1.0, 1.1, 1.25, 1.4]

**Table 4:** Model performance for multi-currency (KRW, USD, INR, and JPY) recognition.

Case	1	2	3	4	5	6
Accuracy (%)	99.76	99.81	99.86	99.95	99.96	99.97
Number of detection errors	49	38	32	8	6	0
Number of classification errors	19	16	6	6	4	6
1st accuracy (%)	99.89	99.92	99.93	99.95	99.95	99.97
2nd accuracy (%)	99.63	99.69	99.80	99.95	99.95	99.97

**Table 5:** Comparison of banknote serial number recognition methods.

Approach	Method	Task	Number of Classes	Detection Accuracy (%)	Classification Accuracy (%)
Handcrafted feature extraction	Zhao et al. [10]	Classification	17	-	95
	Ebrahimzadeh et al. [11]	Classification	17	-	97.25
	Feng et al. [13]	Classification	17	-	99.33
Deep- learning based	Alwzwazy et al. [16]	Classification	17	-	95.7
	Boufenar et al. [17]	Classification	17	-	100
	Zhao et al. [19]	Classification	17	-	99.99
	Wang et al. [21]	Classification	17	-	99.89
	Jang et al. [22]	Classification	17	-	99.92
	Our method	Detection + Classification	37	100	99.97

**Table 6:** Performance comparison of the single-currency dataset-based and multi-currency dataset-based models.

National Currency	Metrics	Single-Currency Model	Multi-Currency Integrated Model
KRW	Accuracy (%)	99.88	99.94
	Number of detection errors	0	0
	Number of classification errors	9	4
	1st accuracy (%)	100	99.94
	2nd accuracy (%)	99.75	99.94
USD	Accuracy (%)	99.94	99.97
	Number of detection errors	5	0
	Number of classification errors	0	2
	1st accuracy (%)	99.95	99.97
	2nd accuracy (%)	99.92	99.97
INR	Accuracy (%)	99.88	100
	Number of detection errors	5	0
	Number of classification errors	3	0
	1st accuracy (%)	99.93	100
	2nd accuracy (%)	99.83	100
JPY	Accuracy (%)	99.80	100
	Number of detection errors	0	0
	Number of classification errors	13	0
	1st accuracy (%)	99.98	100
	2nd accuracy (%)	99.62	100

## Data Availability

Not applicable.
